# Examining Motivation of First-Year Undergraduate Anatomy Students Through the Lens of Universal Design for Learning (UDL): A Single Institution Study

**DOI:** 10.1007/s40670-023-01823-x

**Published:** 2023-07-05

**Authors:** Audrey M. K. Dempsey, Yvonne M. Nolan, Mutahira Lone, E. Hunt

**Affiliations:** 1 Department of Anatomy and Neuroscience, School of Medicine, University College Cork, Cork, Ireland; 2 Department of Occupational Science and Occupational Therapy, School of Clinical Therapies, University College Cork, Cork, Ireland

**Keywords:** Anatomy education, Engagement, Healthcare education, Motivation, Universal design for learning

## Abstract

Motivation is critical for meaningful learning among healthcare students studying anatomy. Learners are highly variable, and it is important to ensure learners are equally supported in the diverse aspects of an anatomy curriculum. The implementation of the educational framework, Universal Design for Learning (UDL), in anatomy curricula could potentially enhance student motivation. The multiple means of engagement principle of UDL refers to the enhancement of motivation among students. This study aimed to identify healthcare students’ motivation levels at the start and end of their anatomy module and whether there was any change in motivation. The Motivated Strategies for Learning Questionnaire (MSLQ) was distributed to gather the self-reported motivation levels of first-year undergraduate medical, dental and occupational therapy (OT) and speech and language therapy (SLT) students studying anatomy at the start of their respective anatomy modules and again at the end of the module. The overall response rate was 74% and 69%, at the start and end of the study, respectively. Responses were analysed by the respective programme of study. Motivation to study anatomy among medical, dental, OT and SLT students ranged from medium to high on the MSLQ at the start of their respective anatomy modules. By the end of the anatomy modules, dental students reported high levels of motivation to study anatomy, whereas motivation among medical, OT and SLT students ranged from medium to high. A change in students’ self-reported motivation levels while studying anatomy was identified. The study emphasises the benefits of UDL and its flexible nature to enhance motivation.

## Introduction 

Motivation is an essential part of students’ academic performance and meaningful learning [1]. It is also an integral element of their career development [2]. There are two types of motivation, intrinsic and extrinsic, and both are known to affect student learning [3]. Intrinsic motivation is characterised as a fundamental satisfaction of completing a task and carrying out the activity for enjoyment and gratification [4], while extrinsic motivation is concerned with external values and accolades, for example, the achievement of high grades [5]. In anatomy education, the absence of either intrinsic or extrinsic motivation has been linked with poor academic performances [6, 7]. The pivotal role of intrinsic motivation in the education and training of healthcare students towards becoming skilled and effective healthcare professionals, through the creation of a positive learning experience, is becoming increasingly recognised [8–11].

Anatomy education has been described as critical for good clinical practice among medical, dental and allied health professionals [12]. However, the study of anatomy requires the understanding and retention of large volumes of information [13]. In recent years, there has been a reduction in the amount of time dedicated to teaching anatomy in many healthcare programmes [14–16], resulting in a focus on students to engage in self-directed learning [17, 18]. Studies have identified an improvement in academic performance among anatomy students as a result of incorporating self-directed learning strategies into curriculum design and delivery [19, 20]. However, motivation plays a crucial role in successful self-directed learning [21]. Thus, understanding student motivation to study anatomy is important for student success.

Many healthcare students struggle with the study of anatomy [22, 23]. Some students find it difficult to remain motivated to study, while others grapple with the relevance of anatomy to their chosen careers [24], and many do not perceive the value of anatomy until they engage in clinical placement [22, 25, 26], typically later in their programme. After exposure to the workplace, many healthcare students develop a newfound appreciation of anatomy [27], but anatomy lectures and practicals are often finished at this stage, and the opportunities to engage with the resources available are missed. Therefore, there is a need to motivate students from the very start of their third-level education and highlight the relevance and value of having a robust knowledge of anatomy. In turn, students require encouragement and guidance on how to become lifelong confident learners who are capable of optimising their learning and becoming efficient learners and healthcare practitioners [28, 29]. All learners have different strengths, and it is important to ensure that they are equally supported and included in the diverse aspects of an anatomy curriculum [4].

### Implementation of Universal Design for Learning

Implementation of the educational framework Universal Design for Learning (UDL) in anatomy curricula could potentially enhance student motivation and engagement among healthcare students studying anatomy. UDL is a framework which guides educators with curriculum design to ensure all learners are accounted for in the one environment [30]. This may be accomplished by merging flexible methods of assessment and teaching for students attending the same tutorial room, lecture hall or laboratory [31]. The three guiding principles of the UDL framework are multiple means of engagement, multiple means of representation and multiple means of action and expression, which are broken down into 31 checkpoints [30]. The framework is already in use in a variety of disciplines including marketing, family and consumer sciences and ecology and in numerous countries such as the USA, South Africa and Canada [32–34]. However, the formal utilisation of UDL in anatomy curricula has yet to be published [35]. Moreover, the use of UDL in anatomy education specifically to enhance motivation has not been identified, discussed nor researched.

The multiple means of engagement principle of the UDL framework refer to the encouragement and enhancement of motivation among students while cultivating enthusiasm for learning [36]. There are numerous theories of motivation including attribution theory [37], social cognitive theory [38] and self-determination theory [39] which form the basis of student motivation questionnaires such as the Motivated Strategies for Learning Questionnaire (MSLQ) [40], used in the current study, to assess the self-reported academic motivation of students, including students in higher education [41].

The aim of this study was to investigate self-reported motivation among first-year undergraduate healthcare students, in a single institution, to study anatomy, and whether there was a change in motivation among the students from the start of their respective anatomy modules to the end of the modules. The study used the MSLQ to address a number of research questions:

1. How motivated are first-year undergraduate medical, dental, OT and SLT students to study anatomy at the start of their third-level education?
2. How motivated are first-year undergraduate medical, dental, OT and SLT to study anatomy at the end of their anatomy module?
3. Is there a change in motivation from the start of the anatomy module to the end among specific healthcare programmes?

The results of this study will be discussed and analysed through the lens of UDL. Opportunities where UDL could have been, and could be, potentially implemented to enhance healthcare students’ motivation to study anatomy will be identified.

## Methods and Materials

### Educational Context and Participants

This prospective cohort study was carried out in University College Cork (UCC), Ireland, with first-year undergraduate students enrolled in either BSc Occupational Therapy, BSc Speech and Language Therapy, MB, BCh, BAO Medicine or BDS Dentistry. Each of these undergraduate healthcare programmes has a compulsory gross anatomy module as a part of the first-year curriculum. The specific content and detail of the respective gross anatomy modules vary between programmes. Each module has a practical element which takes place in the Facility for Learning Anatomy, Morphology and Embryology (FLAME) laboratory where student learning is supported by the inclusion of a number of different methods and materials, including prosections, anatomical models, computer programmes and demonstrations, to consolidate their learning. Students enrolled in each of the healthcare programmes attend approximately 20 h of lectures and 16 h of anatomy practicals per anatomy module. The anatomy modules are supported by the host university’s learning management system (LMS), which provides students’ access to the learning material for their respective lectures and practical sessions.

### Instrument

The MSLQ [40] was used to assess self-reported academic motivation of students. This previously validated and widely used questionnaire was created to measure a number of motivational and self-regulated learning constructs and thus is divided into a motivation section and a learning strategies section [40]. There are 31 items in the academic motivation section of the MSLQ which are grouped into six motivation subscales. The six motivation subscales of the MSLQ (with their MSLQ Scoring Manual Reference Cronbach’s α listed) are “intrinsic goal orientation” (*n* = 4; α = 0.74); “extrinsic goal orientation” (*n* = 4; α = 0.62); “task value” (*n* = 6; α = 0.90); “control of learning beliefs” (*n* = 4; α = 0.68); “self-efficacy for learning and performance” (*n* = 8; α = 0.93) and “test anxiety” (*n* = 5; α = 0.80). The questionnaire is scored on a 7-point Likert scale ranging from 1 is “not at all true of me” to 7 is “very true of me”. Demographic data including the participant’s gender, age and programme of study were collected.

### Data Collection

A physical copy of the MSLQ was distributed by the first author (A.M.K.D) to first-year undergraduate healthcare students studying anatomy during the first week of semester one of the academic year 2019/2020 at the end of an in-person anatomy practical. The same questionnaire was distributed to the same cohorts at the end of their final (in-person) anatomy practical session at the end of their respective anatomy modules. The students had as much time as they required to complete the questionnaire. Informed consent was obtained. Participation by students was entirely voluntary and remained anonymous throughout the process. Ethical approval was obtained from the institutional Social Research Ethics Committee (Log 2019–127). Students under the age of 18 years were excluded as parental consent is required. Only aggregate level data are presented as ethical approval did not allow for identifying and matching students’ responses at the start and end of the anatomy module.

### Statistical Analysis

Data from the MSLQ were analysed using Statistical Package for Social Scientists (SPSS), version 28 (IBM Corp., Armonk, NY). Frequencies and percentages were used to summarise the data. Parametric analyses were used to ensure that this study was comparable to similar studies utilising the MSLQ [42, 43]. Data were analysed using independent *t*-tests to assess changes in the mean responses of the motivation subscales from the start to the end of the anatomy module. Data are reported as mean ± standard deviation (SD). Differences with a *p* value less than 0.05 were considered statistically significant. * denotes statistically significant differences at 0.05. ** denotes statistically significant differences at 0.01. Reliability analysis of each motivation subscale was conducted for the present study and reported as Cronbach’s alpha (α).

## Results

### Demographic Characteristics

The questionnaire was distributed to 221 potential participants both at the start and the end of the study. There were 125 students enrolled in the medical programme and 36 students in the dental programme, and 30 students were enrolled in the occupational therapy and speech and language therapy programmes, respectively. The overall response rate at the start of the study (and respective anatomy modules) was 74% (*n* = 164) and 69% (*n* = 153) at the end of the study. The majority of the participants in this study were female (*n* = 127 and *n* = 105 at the start and at the end of the study, respectively) and aged 18 (*n* = 71 and *n* = 52) and 19 (*n* = 56 and *n* = 61) years. About 20% of participants at the start of the study and at the end were over the age of 20 years. Demographic information from the participating students at the start of the study and again at the end of the study is summarised in Table 1.

**Table 1:** Distribution of participants at the start and end of the study

	**Start**	**End**
Number of participants	164	153
**Gender**		
Male	37 (23%)	48 (31%)
Female	127 (77%)	105 (69%)
**Healthcare programme**		
Medicine	80 (49%)	97 (63%)
Dentistry	26 (16%)	17 (11%)
Occupational therapy	30 (18%)	18 (12%)
Speech and language therapy	28 (17%)	21 (14%)
**Age** (years)		
18	71 (43%)	52 (34%)
19	56 (34%)	61 (40%)
≥ 20	38 (23%)	40 (26%)

### Reliability Analysis

The reliability and internal consistency for each of the subscales for this study is reported alongside the recommended Cronbach’s α value from the MSLQ Scoring Manual [40] (Table 2). The results are considered acceptable as they are in line with the recommended value.

**Table 2:** Reliability analysis of each motivation subscale at the start and end of the anatomy module reported as Cronbach’s α

**Motivation subscale**	Cronbach’s α **Start**	Cronbach’s α **End**	Cronbach’s α [40]
Intrinsic goal orientation	0.7	0.74	0.74
Extrinsic goal orientation	0.55	0.63	0.62
Task value	0.86	0.86	0.90
Control of learning beliefs	0.62	0.7	0.68
Self-efficacy for learning and performance	0.92	0.91	0.93
Test anxiety	0.73	0.73	0.8

The responses from the participants were analysed by programme of study to obtain a measure of motivation among each cohort during the first week of enrolment on the anatomy module and again during the final week of enrolment. The breakdown of the self-reported mean scores from the motivation subscales of the MSLQ at the start and end of the module from each cohort is reported in Table 3.

**Table 3:** MSLQ mean motivation subscale scores of dental and medical OT and SLT students at the start and end of studying an anatomy module

***Dentistry***					
	**Start**	**End**	Independent *t*-test	*p* value	Change in motivation
**Motivation subscale**	(*n* = 26) Score (± SD)	(*n* = 17) Score (± SD)			
Intrinsic goal orientation	5.36 (± 0.79)	5.05 (± 0.97)	1.105	0.278	-
Extrinsic goal orientation	5.5 (± 0.88)	5.53 (± 0.96)	−.102	0.919	-
Task value	6.17 (± 0.72)	6.13 (± 0.78)	.166	0.869	-
Control of learning beliefs	6.08 (± 0.54)	6.12 (± 0.52)	−.246	0.807	-
Self-efficacy for learning and performance	5.13 (± 0.77)	5.52 (± 0.88)	−1.502	0.143	-
Test anxiety	4.67 (± 1.1)	5.06 (± 1.1)	−1.139	0.263	-
***Medicine***					
	**Start**	**End**	Independent *t*-test	*p* value	Change in motivation
**Motivation subscale**	(*n* = 80) Score (± SD)	(*n* = 97) Score (± SD)			
**Intrinsic goal orientation**	**5.38 (± 0.83)**	**4.92 (± 1.07)**	**3.269**	0.001**	**↓**
Extrinsic goal orientation	5.36 (± 0.94)	5.17 (± 1.06)	1.271	0.206	-
**Task value**	**6.28 (± 0.71)**	**5.91 (± 0.86)**	**3.125**	0.002**	**↓**
Control of learning beliefs	5.71 (± 0.89)	5.75 (± 0.9)	-.281	0.779	-
Self-efficacy for learning and performance	5.08 (± 1.0)	4.99 (± 1.0)	.601	0.549	-
**Test anxiety**	**4.03 (± 1.19)**	**4.57 (± 1.22)**	**-3.007**	0.003**	**↑**
***Occupational therapy (OT)***					
	**Start**	**End**	Independent *t*-test	*p* value	Change in motivation
**Motivation subscale**	(*n* = 30) Score (± SD)	(*n* = 18) Score (± SD)			
Intrinsic goal orientation	4.36 (± 1.11)	4.44 (± 0.91)	−.292	0.771	-
Extrinsic goal orientation	5.07 (± 1.03)	5.36 (± 0.85)	−1.075	0.289	-
Task value	5.68 (± 0.96)	5.67 (± 0.88)	.037	0.971	-
Control of learning beliefs	5.49 (± 0.91)	5.32 (± 1.22)	.519	0.608	-
Self-efficacy for learning and performance	4.08 (± 0.96)	4.15 (± 1.04)	−.225	0.824	-
Test anxiety	5.01 (± 1.31)	5.01 (± 1.33)	−.011	0.991	-
***Speech and language therapy (SLT)***					
	**Start**	**End**	Independent *t*-test	*p* value	Change in motivation
**Motivation subscale**	(*n* = 28) Score (± SD)	(*n* = 21) Score (± SD)			
Intrinsic goal orientation	4.49 (± 0.77)	4.5 (± 0.97)	−.035	0.972	-
Extrinsic goal orientation	5.23 (± 0.89)	5.54 (± 0.79)	−1.261	0.214	-
Task value	5.51 (± 0.75)	5.33 (± 0.86)	.791	0.433	-
**Control of learning beliefs**	**5.55 (± 0.77)**	**5.94 (± 0.52)**	**−2.139**	0.038*	**↑**
Self-efficacy for learning and performance	4.38 (± 0.81)	4.53 (± 0.82)	−.637	0.528	-
Test anxiety	4.93 (± 0.92)	5.26 (± 0.7)	−1.429	0.160	-

Motivation subscales scores ranged from 1 = “not at all true of me” and 7 = “very true of me”

*p < 0.05; **p < 0.001

### Motivation at the Start of the Anatomy Module

Dental students’ motivation at the start of their anatomy module ranged from 4.67 to 6.17. The lowest reported mean value on the Likert scale was for the subscale “test anxiety”, and the highest value was for the motivational subscale “task value”. The lowest reported mean value on the Likert scale among medical students at the start of their anatomy module was 4.03. This was for the subscale “test anxiety”. The highest value was 6.28 and was for the motivational subscale “task value”. The lowest reported mean value on the Likert scale among OT students at the start of their anatomy module was 4.08 for the subscale “self-efficacy for learning and performance”. The highest value was 5.68 and was for the motivational subscale “task value”. Self-reported motivation among SLT students at the start of their anatomy module ranged from 4.38 to 5.55. The lowest reported mean value on the Likert scale was for the subscale “self-efficacy for learning and performance”, and the highest value was for the motivational subscale “control of learning beliefs”.

### Motivation at the End of the Anatomy Module

The lowest self-reported motivation mean value among dental students was 5.05 for “intrinsic goal orientation”. The “task value” subscale remained the highest value on the Likert scale with a score of 6.13. Medical students lowest self-reported motivation mean value was 4.57 for “test anxiety”. The “task value” subscale remained the highest value on the Likert scale with a score of 5.91. The lowest mean value of OT students self-report motivation was 4.15 for “self-efficacy for learning and performance”. The “task value” subscale remained the highest mean value on the Likert scale with a score of 5.67. The lowest mean value among SLT students was 4.50 for “intrinsic goal orientation”. The “control of learning beliefs” subscale remained the highest mean value on the Likert scale with a score of 5.94.

### Change in Motivation Over the Course of the Anatomy Module

There were no significant differences in mean motivation scores obtained from the dental or OT students between the start and the end of their respective anatomy modules. There was a significant decrease in “intrinsic goal orientation” (*p* = 0.001) and “task value” (*p* = 0.002) in the cohort of first-year medical students between the start and end of their anatomy module. “Test anxiety” in these medical students between the start and end of the module was significantly increased (*p* = 0.003). The only MSLQ subscale where mean motivation scores were significantly different between the start and the end of the study in SLT students was “control of learning beliefs”. The SLT students reported a significant increase (*p* = 0.038) in “control of learning beliefs” at the end of the anatomy module compared to the start (Table 3).

## Discussion

The task of stimulating students’ motivation to learn is a fundamental challenge in education [44]. Understanding how healthcare students are motivated to study anatomy can potentially influence curriculum design to advance self-directed learning and academic performance [45]. The healthcare students in this study were first-year undergraduates, who were predominately aged 18 and 19 years old. These students were in the process of transitioning from secondary to tertiary education. Research has shown that students beginning their journey through third-level education require guidance and support to become motivated to study while simultaneously navigating their new routines and environments [46, 47]. Additionally, approximately 20% of participants are over the age of 20 years. This corresponds to the typical allotment of places for mature students in undergraduate programmes [48].

Using the method of Cho et al. (2017), the subscale scores were categorised as low (1 to < 2.5), medium (2.5 to < 5) and high scores (5 to < 7) on the Likert scale. This study identified that motivation levels among first-year undergraduate healthcare students ranged from medium to high [49] on the MSLQ Likert scale at the start of their respective anatomy modules. At the end of the anatomy module, only dental students reported high motivation levels to study anatomy, whereas at the same time point, motivation levels to study anatomy among medical, OT and SLT students ranged from medium to high on the Likert scale. This could perhaps be due to the anatomy module for dental students spanning two semesters, while the anatomy modules for the other cohorts all occurred in semester one. There is little research regarding the impact of semesterisation on academic performance or motivation [50]. Detailed analysis of the results identified a change in motivation between the start and end of studying an anatomy module among each of the four cohorts (Table 3). Changes in self-reported motivation may be cause for revaluation of the design and delivery of anatomy curricula and more specifically, a focus on how students are, or could be, motivated and engaged in the process of learning.

Intrinsic and extrinsic motivation have been reported to play a role in student learning [44]. In this study “extrinsic goal orientation” was high on the Likert scale across all four cohorts at the start of their anatomy module and remained high at the end of the module. Similarly, Zilundu et al. (2022) reported high “extrinsic goal orientation” scores among medical students studying anatomy, at the end of their module. In the current study, there was no significant change in either intrinsic or extrinsic motivation for dental, OT or SLT students. “Intrinsic goal orientation” ranged from medium to high, on the MSLQ Likert scale, across all four cohorts at the start of the anatomy module. However, at the end of the anatomy module, only dental students reported high “intrinsic goal orientation”. Although the mean score for this motivational subscale remained high among dental students, their motivational levels did decrease over the course of the module. The motivational levels for medical, OT and SLT students were medium on the Likert scale at the end of the anatomy module. These results are similar to those reported by Abdel Meguid et al. (2020) who distributed the MSLQ to first-year chiropractic and dental students, studying anatomy, at the end of their module. The current study reported a decrease in “intrinsic goal orientation” among medical students, which suggests that they may have lost interest in anatomy as the module progressed. This becomes worrisome when the integral role of anatomy in their chosen careers is considered [51]. The relevance of anatomy may need to be repeatedly emphasised to students enrolled in all four healthcare programmes during their learning to help sustain interest, although there was a marginal increase in “intrinsic goal orientation” among OT and SLT students. McNamara and Nolan reported that incorporating specific applied anatomy activities to the curriculum to emphasise anatomy as clinically important, made learning more interesting and engaging for medical students [52]. This is in line with the checkpoint 7.2 “Optimise relevance, value, and authenticity” of the UDL pedagogical framework [30].

Task value refers to an individual’s opinion of the importance of an activity [53]. The mean score for the “task value” subscale was reported as high across all four cohorts both at the start of the anatomy module and also at the end. These results are on par with those reported by first-year students enrolled in a medical programme in 2017 and 2019 [45]. However, the results for “task value” in the current study are higher than the scores reported by first-year chiropractic and dental students in a previous study [43]. A recent study carried out by Zilundu et al. (2022) found that a driving factor of medical students’ motivation to study anatomy was the importance and value of the subject matter. The self-reported “task value” among medical students in the current study decreased significantly between the start and end of studying the anatomy module. This suggests that the medical students did not value the study of anatomy by the end of their module. It could be argued that students should have a heightened opinion of the importance of anatomy at this stage. Perhaps the incorporation of teaching strategies which align with the UDL checkpoint 7.2 “Optimise relevance, value and authenticity” [30] would enhance students’ understanding of why anatomy is important to study. For example, emphasising the clinical significance of certain structures or describing scenarios when they would have to recall certain anatomical information could optimise “task value” among learners. Similarly, educators could introduce gamification, to highlight the relevance and value of anatomy, as Dugnol-Menéndez et al. described an increase in learning motivation among OT students when they introduced gamification to the design of their anatomy curriculum [54].

At the start of the anatomy module, the mean score for the MSLQ subscale “control of learning beliefs” was high for all four cohorts, and the mean score remained high at the end of the anatomy module. Dental students had a higher mean Likert score for the subscale “control of learning beliefs” at the end of their module compared to other dental students studying anatomy in first year [43], and the medical students were on par with other medical students’ self-reported “control of learning beliefs” at the end of the module [45]. The mean score for the motivation subscale “control of learning beliefs” significantly increased in SLT students between the start and end of the study suggesting that SLT students believed that if they were to study in appropriate ways, then they would be able to master the learning material. It also implies that as SLT students progressed through the anatomy module they became significantly more aware of their capabilities. It could be speculated that this was a result of educators incorporating teaching strategies which encouraged the students to monitor their progress, have confidence in their abilities and engage in self-reflection, all of which align with the UDL checkpoint 6.4 “Enhance capacity for monitoring progress”, checkpoint 9.2 “Promote expectations and beliefs that optimise motivation” and checkpoint 9.3 “Develop self-assessment and reflection”. In contrast, there was a decrease in “control of learning beliefs” among OT students from the start of their module to the end. This suggests that OT students were unable to sustain the belief in their own learning abilities which they possessed at the beginning of their journey into third-level education. There are a number of reasons why this may have occurred, including situational factors such as the impending summative examinations or feelings of being overwhelmed by the amount of learning material which they must study. The UDL checkpoints 7.1 “Optimise individual choice and autonomy”, 8.2 “Vary demands and resources to optimise challenge” and checkpoint 9.2 “Facilitate personal coping skills and strategies” are reported to promote self-belief among students by recruiting interest and sustaining effort, respectively [30].

Learner’s self-efficacy beliefs drive their level of motivation [38]. Tembo and Ngwira (2016) concluded that self-regulation was closely related to self-efficacy beliefs in anatomy education [55]. In the present study, students “Self-efficacy for learning and performance” ranged from medium to high on the Likert scale at the start of their anatomy module for all cohorts. The range of scores remained between medium and high on the Likert scale at the end of the anatomy module. These results are lower than self-reported self-efficacy among medical students studying anatomy in 2017 and 2019 [45]. There was no significant change in the mean score for self-efficacy for learning and performance in any cohort between the start and the end of the anatomy module.

Test anxiety has been found to be negatively related to academic performance [56, 57]. In this study, “test anxiety” was reported as high, as defined by Cho et al. (2017), by dental, OT and SLT students at the end of their anatomy module. There was no statistically significant change in “test anxiety” among these three cohorts. Medical students self-reported “test anxiety” at the end of their anatomy module was also high when compared to similar studies [45, 56]. Self-reported “test anxiety” was significantly increased in medical students at the end of the module, compared to the start of the module, which coincided with the approach of the examination period. Test anxiety is made up of two elements, worry and emotionality [58, 59]. Warnecke et al. (2019) reported that guiding students on the use of effective learning strategies or self-regulatory skills reduced test anxiety among third-level students [60]. Hence, educators could consider the implementation of UDL in the design and delivery of their anatomy curriculum to introduce learners to multiple learning strategies so that students may be able to choose their optimal and preferred method of studying [30]. Zilundu et al. (2022) concluded that anatomy educators should be aware of their students’ motivations and learning strategies so that they may encourage self-regulated and lifelong learning traits. The UDL guideline 9, “Provide options for self-regulation” is broken down into the following checkpoints: checkpoint 9.1 “Promote expectations and beliefs that optimise motivation”, 9.2 “Facilitate personal coping skills and strategies” and 9.3 “Develop self-assessment and reflection” [30]. Similar to the current study, Bischofsberger et al. (2021) reported an increase in test anxiety in medical students at the end of an anatomy module compared to the start. Specifically, they found that the percentage of first-year medical students reporting test anxiety increased from 15 to 25% [61]. By incorporating teaching strategies which align with UDL in the design and delivery of curricula, anatomy educators would afford students the opportunities to increase their confidence and become more comfortable with learning the anatomy material. In particular, educators could incorporate teaching strategies such as the inclusion of self-assessment activities which allow students to monitor their progress, reflect on their understanding and gain immediate feedback. However, more research is required to understand precisely how the inclusion of UDL could alleviate test anxiety among students studying anatomy, particularly those enrolled in dental, OT or SLT programmes.

To summarise, the changes in self-reported motivation among the participants suggest a revaluation of the design and delivery of third-level anatomy curricula may be timely. Future research could focus on how students are, or could be, motivated and engaged in the study of anatomy. The inclusion of teaching strategies which align with the UDL framework, such as emphasising the relevance of anatomy, promoting self-belief among learners and providing students with opportunities for self-assessment, monitoring of progress and reflection, could potentially enhance and sustain motivation among healthcare students studying anatomy.

### Limitations

The present study used the MSLQ which is a self-report questionnaire. Although this questionnaire has been validated, it can only measure motivation among the healthcare students as accurately as the students’ own perceptions allow. Additionally, this study was conducted with first-year undergraduate students in a single institution, thus limiting generalisability of the findings. Another limitation of this study was that the means results gathered at the start of the anatomy modules and at the end of the respective modules cannot be linked to an individual student due to limits of institutional ethical approval. Thus, it cannot be stated whether it was the exact same students who completed both questionnaires. Statistical changes were reported to a greater degree among the medical cohort when compared to the dental, OT and SLT cohorts. This may be due to the higher number of participants enrolled in the medical programme and thus the increased power for statistical analysis. Furthermore, the reported increase in test anxiety among participants at the end of their anatomy module may, in part, be due to the fact that formal examinations commenced in the forthcoming weeks.

## Conclusion

This study identified a change in medical, dental, OT and SLT students’ self-reported motivation while studying anatomy. The findings highlight the benefits of UDL and its flexible nature to enhance motivation and promote learning. Furthermore, it identifies areas where theories of motivation align with the UDL checkpoints.

## Data Availability

The datasets generated and analysed during the current study are available from the corresponding author on reasonable request.
